# Modulation of GABAergic dysfunction due to *SCN1A* mutation linked to Hippocampal Sclerosis

**DOI:** 10.1002/acn3.51150

**Published:** 2020-08-05

**Authors:** Gabriele Ruffolo, Katiuscia Martinello, Angelo Labate, Pierangelo Cifelli, Sergio Fucile, Giancarlo Di Gennaro, Andrea Quattrone, Vincenzo Esposito, Cristina Limatola, Felice Giangaspero, Eleonora Aronica, Eleonora Palma, Antonio Gambardella

**Affiliations:** ^1^ Department of Physiology and Pharmacology Istituto Pasteur‐Fondazione Cenci Bolognetti University of Rome Sapienza Rome Italy; ^2^ IRCCS Neuromed Pozzilli Isernia Italy; ^3^ Institute of Neurology University Magna Græcia Catanzaro Italy; ^4^ Institute of Molecular Bioimaging and Physiology of the National Research Council Catanzaro Italy; ^5^ Department of Biotechnological and Applied Clinical Sciences (DISCAB) Amsterdam UMC, University of L'Aquila L'Aquila Italy; ^6^ Department of (Neuro)Pathology Amsterdam UMC, University of Amsterdam Amsterdam the Netherlands; ^7^ Stichting Epilepsie Instellingen Nederland Heemstede the Netherlands

## Abstract

We compared GABAergic function and neuronal excitability in the hippocampal tissue of seven sporadic MTLE patients with a patient carrying a *SCN1A* loss‐of‐function mutation. All had excellent outcome from anterior temporal lobectomy, and neuropathological study always showed characteristic hippocampal sclerosis (Hs). Compared to MTLE patients, there was a more severe impairment of GABAergic transmission, due to the lower GABAergic activity related to the Na_V_1.1 loss‐of‐function, in addition to the typical GABA‐current rundown, a hallmark of sporadic MTLE. Our results give evidence that a pharmacological rescuing of the GABAergic dysfunction may represent a promising strategy for the treatment of these patients.

## Introduction

Mesial temporal lobe epilepsy (MTLE) with hippocampal sclerosis (Hs) represents the most common type of focal drug‐resistant epilepsy.^1^ Although MTLE is now routinely treated with neurosurgery, over a third of MTLE patients do not achieve seizure freedom,^2^ and surgery can have important adverse consequences. Better treatment options, or even prevention, of MTLE and Hs are therefore needed, but rational therapy remains elusive because their causes remain unclear. A large body of evidence indicates the biologic processes that are relevant in the pathogenesis of Hs include glial activation, immune response, synaptic transmission, signal transduction, ions transport, and synaptic plasticity.^3^, ^4^ In the hippocampus, GABAergic inhibitory dysfunction mostly contributes to hyperexcitability in MTLE with Hs.^5^, ^6^, ^7^

Of interest, recent studies illustrate *SCN1A* involvement in the epileptogenic neuronal network underlying MTLE associated with Hs.^8^ Accordingly, we already illustrated that MTLE with febrile seizures may be part of the epileptic phenotype encountered in a large family carrying a *SCN1A* mutation.^9^, ^10^ This M145T mutation of *SCN1A* cosegregated in all affected individuals of a large family affected by simple febrile seizures (FS), three of whom later developed MTLE.^10^ Most important, the M145T mutation is located in a highly conserved amino acid in the first transmembrane segment of domain I of the *SCN1A*, and functional studies in mammalian cells demonstrated that the M145T mutation causes a 60% reduction of current density and a 10 mV positive shift of the activation curve.^9^ More recently, one family member with refractory MTLE and Hs has benefited from surgery, and now we wish to report the extensive neurophysiological study from the resected hippocampal tissue of this patient, in comparison with seven sporadic patients with refractory MTLE and Hs. We believe that this study may provide important new directions to the physiopathological comprehension of MTLE with Hs and FS, and ultimately may pave the road to develop new therapeutic approaches in MTLE.

## Patients and Methods

### Patients

The clinical data of seven sporadic MTLE patients with Hs, whose hippocampal tissues were used for electrophysiological recordings, are described in detail in Supplemental Material and summarized in Table S1. The patient (mutated, mNa_V_1.1) carrying a loss‐of‐function missense mutation (M145T) of *SCN1A* is a 31‐year‐old man with MTLE, whose electroclinical data were reported in detail elsewhere.^9^, ^10^ He had simple FS every 3–6 months from the age of 8 months until 70 months. At the age of 13 years, he began to have afebrile seizures characterized by behavioral arrest, some lip smacking and gestural automatisms, or nocturnal secondary generalized motor seizures. Over the years, he also began to experience epigastric auras with some experiential phenomena prior to the loss of awareness. Since seizures became refractory to antiepileptic drugs (AEDs) that included oxcarbazepine, phenobarbital, topiramate, and valproate, and he underwent an extensive presurgical evaluation. Interictal awake and sleep EEG recording showed bilateral mesiotemporal epileptiform spikes, which strongly predominated (>80%) on the right side. On intensive video‐polygraphic monitoring, several typical seizures with onset in the right midinferomesial temporal region were recorded. Brain MRI revealed right *Hs* and, at the age of 27, he underwent surgery, in the form of right anterio‐temporal lobectomy. Histopathological findings disclosed type 2 Hs with the characteristic pattern of predominant neuronal loss in area CA1.^3^, ^4^ Since surgery, in the last 50 months, he has only had non‐disabling auras (Engel’s outcome classification Class 1B), and he has been taking a dual therapy with carbamazepine plus valproate.

### Electrophysiology

See Supplemental Material for details. The study was approved by the local Ethics Committees.

## Results

### Patch‐clamp experiments in human slices

**Figure 1:**
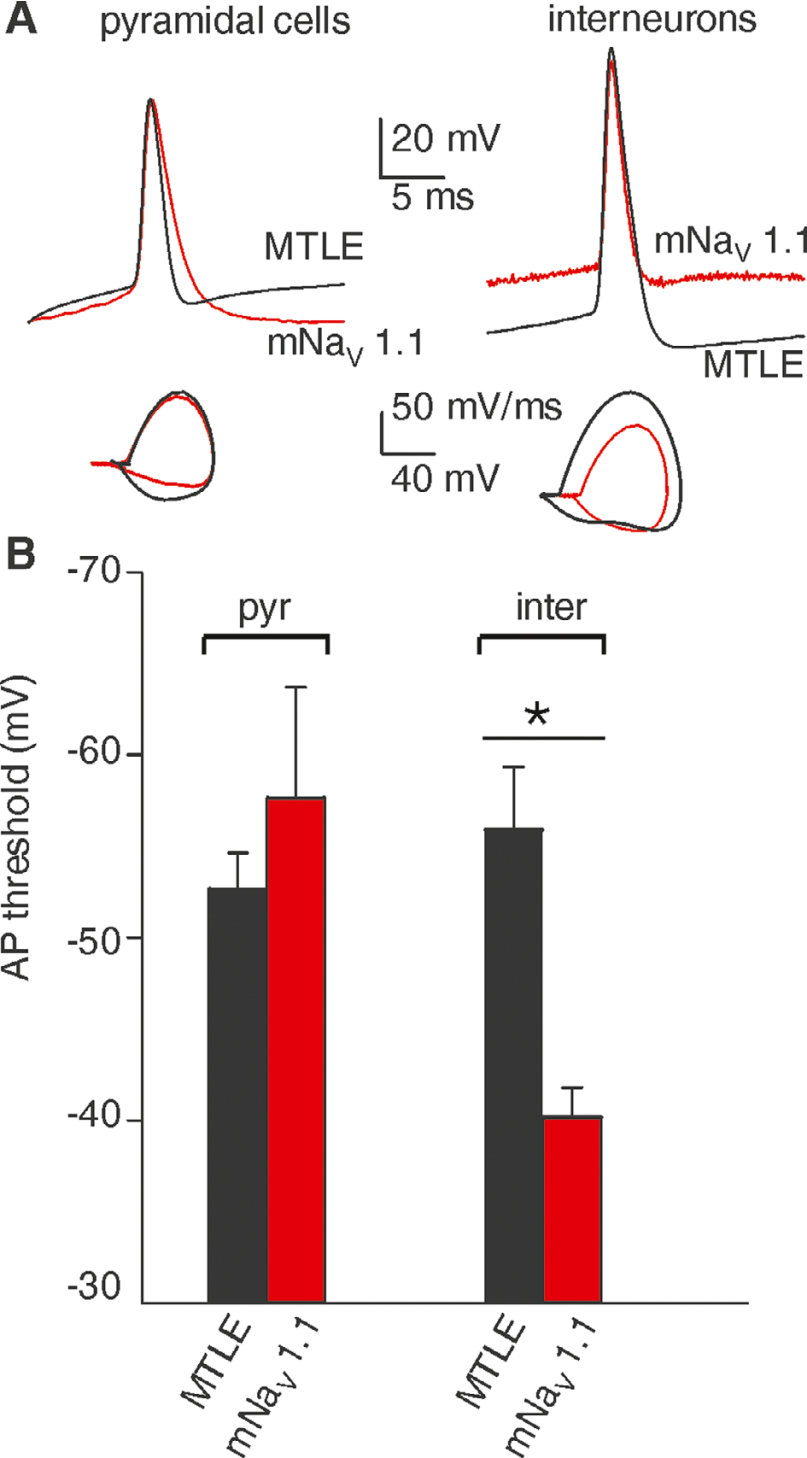
mNaV 1.1 patient exhibits depolarized action potential (AP) threshold in hippocampal interneurons but not in pyramidal cells. (A) Left top, superimposed AP traces recorded from one pyramidal neuron of the mNa_V_ 1.1 patient (red trace) and from one MTLE patient (black trace, #5); left bottom, superimposed phase‐plane plot obtained from APs showed on top. Right top, superimposed AP traces recorded from one interneuron of mNa_V_ 1.1 patient (red trace) and from the same MTLE patient (black trace); right bottom, superimposed phase‐plane plot obtained from APs showed on top. (B) Bar graph representing the mean action potential threshold values recorded from pyramidal neurons (pyr; n = 10) and interneurons (inter; n = 5) in resected hippocampi of patients with MTLE (black bars, #4‐8, Table S1) and from pyramidal cells (pyr; n = 5) and interneurons (inter; n = 3) of mNa_V_ 1.1 patient (red bars). *P = 0.044; One‐way ANOVA Test, and post hoc Holm–Sidak test.

To highlight the functional implications of mNa_V_ 1.1 expression, patch‐clamp experiments were performed on pyramidal cells and interneurons of the resected hippocampus of the mNa_V_1.1 patient, comparing the results with those obtained from sporadic MTLE patients (patients, # 4‐8, Table S1). Current steps (100–150 pA, 500 ms duration) applied to the cells were able to elicit one or more action potentials (APs), with pyramidal neurons from all patients exhibiting APs with similar characteristics, in particular with no change in AP threshold (MTLE: −53 ± 3 mV; n = 10; # 4‐8, Table S1, mNa_V_ 1.1 patient: −53 ± 3 mV; n = 5; *P* = 0.64; Fig. 1A,B). By contrast, three interneurons from the mNa_V_1.1 patient showed a significantly depolarized AP threshold (−40 ± 3 mV) in comparison to interneurons from MTLE patients (−58 ± 12 mV, n = 5; # 4‐8, Table S1; *P* = 0.04; Fig. 1A,B, see Table S2 for electrophysiological parameters). These data suggest that mNa_V_1.1 expressing interneurons may have a reduced intrinsic excitability as expected from M145T mutation of *SCN1A*.^9^

### GABA currents rundown

**Figure 2:**
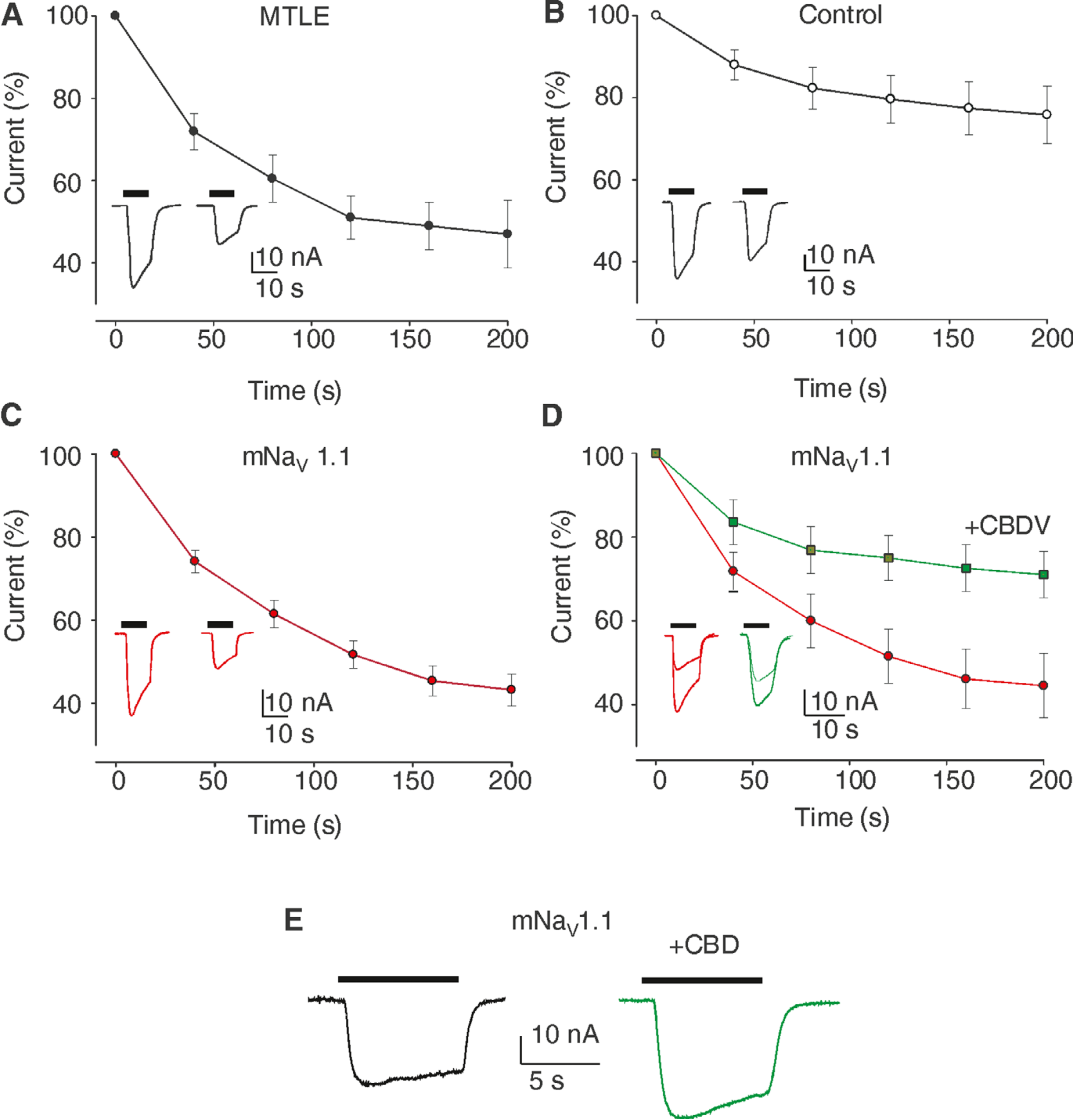
mNaV 1.1 patient exhibits a GABA current rundown similar to MTLE hippocampal tissues. (A) Oocytes injected with hippocampal membranes of MTLE patients (n = 33; I_max_ = 40.5 ± 20 nA; #1,2, 4,5 Table S1). Black dots (●) represent the amplitude of consecutive GABA currents as % of the first response (GABA 500 µM). Data points represent means ± SEM. In this and following panels all the currents are normalized to the first current (I_max_) and black horizontal bars represent the timing of GABA application. In all experiments, the holding potential was −60 mV. *Inset:* Representative current traces elicited by the first and sixth GABA application (500 µM, horizontal bar); (B) Oocytes injected with hippocampal membranes from a non‐epileptic control (n = 10; I_max_ _=_ 49.5 ± 17.3 nA). White dots (○) represent the amplitude of consecutive GABA currents as % of the first response (GABA 500 µM). *Inset*: Representative current traces as in (A). (C) Oocytes injected with hippocampal membranes from mNa_V_ 1.1 patient (n = 19; I_max_ = 30.8 ± 5.7 nA). Red dots (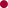) represent the amplitude of consecutive GABA currents as % of the first response (GABA 500 µM). *(Inset)* Representative current traces as in (A). Note the current rundown similar to MTLE patients. (D) Oocytes injected with hippocampal membranes from the mNa_V_ 1.1 patient before and after CBDV treatment. Red dots (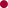) represent the amplitude of consecutive GABA currents as % of the first response (GABA 500 µM) while the green squares 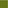) represent the amplitude of consecutive GABA currents on the same cells after 2 hours of incubation with CBDV 50 nM. Data points represent means ± SEM [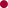) I_max_ = 30.7 ± 8.0 nA; (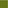) I_max_ = 27.8 ± 7 nA)]. *Inset*: Representative GABA current traces as in (A), before (red traces) and after 50 nM CBDV application (green traces) as indicated. *P* = 0.002, n = 19 by Shapiro–Wilk test and Student's t‐test. (E) Representative GABA current traces evoked by 50 µM GABA in one oocyte of 13 injected with membranes of mNa_V_ 1.1 before (black trace) and after CBD co‐application (5 µM, green trace).

To investigate how GABA currents respond in mNa_V_1.1 patient’s hippocampus, we performed microtransplantation experiments injecting oocytes with hippocampal tissues from sporadic MTLE patients (without known genetic mutations and with Hs, Fig. 2A), one control individual (Fig. 2B) and mNa_V_ 1.1 patient (Fig. 2C‐E). The results confirmed that a strong, pathological GABA‐current rundown is present in drug‐resistant MTLE hippocampi (49.0 ± 9.1%; n = 33; patients #1,2,4,5 Table S1; Fig. 2A), whereas the same phenomenon was absent in the control (75.8 ± 7.0 %; n = 10; # 3, Table S1; Fig. 2B) as previously shown.^5^, ^6^, ^7^

Interestingly, the sclerotic hippocampus of mNa_V_ 1.1 patient also showed a GABAergic rundown (43.2 ± 3.1 %; n = 19; Fig. 2C) similar to sporadic MTLE patients. This current rundown was mitigated by pretreatment of 2 hours with endogenous factors, as BDNF, or exogenous agents, as the cannabis derivative cannabidivarine (CBDV, Fig. 2D; Table S3) showing again a strong similarity with data previously published for MTLE patients.^5^, ^11^

Interestingly, we found that acute co‐application of cannabidiol (CBD, 5 µM, GABA 50 µM), which has been reported to be clinically effective in Dravet syndrome,^12^ potentiated both GABA‐current amplitude from mNa_V_ 1.1 patient (I_GABA_; +29.8 ± 4.1 %; n = 13; Fig. 2E, Table S3) and from sporadic MTLE patients (I_GABA_; +27.5 ± 8.8 %; n = 22; #1,2, Table S3).^13^

## Discussion

The novelty of our study is that we performed electrophysiological experiments from an exclusive hippocampal tissue obtained from a patient with MTLE and Hs, who also carried the M145T *SCN1A* loss‐of‐function mutation. Compared to hippocampal tissue from MTLE patients, there was a more severe impairment of GABAergic transmission, due to the lower GABAergic activity related to the Na_V_1.1 loss‐of‐function, in addition to the characteristic GABA‐current rundown, a hallmark of MTLE.^5^, ^6^, ^7^, ^11^

We recorded valuable APs on both pyramidal neurons and interneurons from mNa_V_ 1.1 and five (of seven) sporadic MTLE patients. In all of them, the histopathological study showed the features of Hs related to MTLE, with the characteristic pattern of neuronal loss in CA1 (see Table S1).^3^, ^4^ Nonetheless, only in hippocampal mNa_V_ 1.1 slices, AP threshold was more depolarized in interneurons than in pyramidal neurons, reflecting a reduced excitability of GABAergic interneurons related to *SCN1A* mutation.^14^, ^15^ In spite of the limited number of recordings on mNav 1.1 interneurons, this type of AP responses paralleled the functional data obtained from cells transfected with M145T mutated cDNA.^9^

Because of the limited quantity of fresh tissue from the patient and technical difficulty of recordings from acute hippocampal slices, part of the tissue was snap‐frozen and used for recordings in microtransplanted oocytes.^6^, ^16^ Notably, we found a GABA current rundown that was very similar to the one recorded from hippocampi of sporadic MTLE patients.^5^, ^6^, ^7^, ^11^ The GABA current rundown is considered an electrophysiological dysfunction of refractory human MTLE,^5^, ^6^, ^7^ which may explain, at least in part, the hyperexcitability underlying the ictogenesis in these patients. Interestingly, we previously showed in epileptic rats that GABA current rundown arises from the hippocampal region (during status epilepticus) then spreading to temporal cortex in the chronic phase of seizures.^7^, ^17^

We already emphasized that the pharmacological modulation of the GABAergic impairment (i.e., GABA current rundown) underlying MTLE may represent an interesting therapeutic approach in pharmacoresistant MTLE.^17^ In this way, it is not surprising that BDNF could ameliorate the GABA current rundown both in the mNa_V_ 1.1 and sporadic MTLE patients, as previously reported for both refractory human MTLE^5^ and preclinical animal models of MTLE.^7^ Notably, CBDV continues to show its potential in recovering the mNav 1.1 GABAergic rundown as previously shown for MTLE.^11^ Furthermore, CBD was capable to increase GABA current amplitude in mNa_V_ 1.1 and MTLE patients, as reported in Dravet syndrome and tuberous sclerosis complex.^12^, ^13^, ^18^ Altogether our results emphasize the role of cannabis derivatives as treatment for refractory epilepsies.

In our patient, the overall clinical, pharmacological, and neuropathological findings were consistent with those observed in sporadic MTLE with Hs, including the excellent outcome from anterior temporal lobectomy. In this way, our findings reinforce the belief that known genetic defect, which is present diffusely in the brain, do not necessarily preclude a good prognosis following epilepsy surgery, if surgery is a reasonable option based on the concordance of other data during presurgical evaluation. It cannot be excluded, however, that the milder loss‐of‐function impairment of SCN1A related to the M145T mutation could also influence the better postsurgical outcome.

Important, as previously reported in Dravet syndrome and tuberous sclerosis complex,^13^, ^18^ CBD was capable to increase GABA current amplitude in mNa_V_ 1.1 and MTLE patients, that was recently approved as treatment for Dravet and Lennox–Gastaut syndromes.^12^ Thus, in accordance with the “interneuron hypothesis,”^14^ our findings support the assumption that seizures in mNa_V_ 1.1 patient could arise from a minor GABA release from interneurons together with a reduction of GABAergic postsynaptic efficacy after repetitive stimulation (i.e., rundown). Major therapeutic implication of these findings is that by rescuing the GABAergic inhibitory activity it is possible to improve the clinical outcome of this kind of patients. In this way, this study ultimately may pave the road to develop new antiepileptic approaches in epileptic patients with Hs.

## Conflicts of Interest

None of the authors has any conflict of interest to disclose.

## Supporting information


**MTLE Patients.** Detailed description of patients.
**Table S1.** Clinical characteristics and neurophysiological findings of MTLE patients.
**Electrophysiology.** Patch‐clamp in human slices; Membrane Preparation, Injection Procedure, and voltage‐clamp Recordings in Oocytes.
**Table S2.** Electrophysiological parameters in patch‐clamped human neurons.
**Table S3.** Effects of pharmacological agents on mNa_V_1.1 and MTLE patients.
**Figure S1.** Firing of hippocampal interneurons.
**Statistics.** Detailed description of the statistical analysis.
