# Supplementary material for: Modulation of GABAergic dysfunction due to SCN1A mutation linked to Hippocampal Sclerosis
Source: Ann Clin Transl Neurol. 2020 Aug 5;7(9):1726–31. doi: 10.1002/acn3.51150 (PMC7480916; doi:10.1002/acn3.51150)
Supplement: Supplementary file 1 — MTLE Patients. Detailed description of patients. Table S1. Clinical characteristics and neurophysiological findings of MTLE patients. Electrophysiology. Patch‐clamp in human slices; Membrane Preparation, Injection Procedure, and voltage‐clamp Recordings in Oocytes. Table S2. Electrophysiological parameters in patch‐clamped human neurons. Table S3. Effects of pharmacological agents on mNaV1.1 and MTLE patients. Figure S1. Firing of hippocampal interneurons. Statistics. Detailed description of the statistical analysis. [file ACN3-7-1726-s001.docx]

**Modulation of GABAergic dysfunction due to *SCN1A* mutation linked to Hippocampal Sclerosis**

**Supplemental material**

**MTLE Patients**

The clinical cases and controls included in this study were selected from the Departments of Neuropathology of the University Medical Center (UMC, University of Amsterdam) and the Neuromed Neurosurgery Center for Epilepsy (Pozzilli, Italy). The clinical characteristics derived from the patients’ medical records are summarized in Table S1. All patients underwent presurgical evaluation with non-invasive tests. Patients who underwent implantation of strip and/or grid electrodes for chronic subdural invasive monitoring before resection were excluded from the study. In MTLE patients, the surgery consisted of an extensive temporal lobectomy (ETL) including microsurgical resection of the amygdala and parahippocampal gyrus and en-bloc excision of the hippocampal formation. The interventions differed in the extent of the neocortical resection. Nondominant ETL included excision of approximately 4 –7 cm or more cm of the superior, middle and inferior temporal gyrus, depending on intraoperative EEG measurements, whereas dominant ETL included excision of approximately 3-7 cm of the superior, middle and inferior temporal gyrus, depending on language mapping and intraoperative EEG measurements. The predominant seizure types were medically intractable focal impaired awareness seizures (FIAS), and all patients had seizures which were resistant to maximal doses of different anti-epileptic drugs (Table S1). Epilepsy duration was calculated as the interval in years from age at seizure onset to age at surgery; no patients included in our series had seizures in the 24 h before surgery. Patients #1-2, 4-8 (Table S1) had hippocampal sclerosis (Hs) with predominant neuronal loss in CA1 (Hs, ILAE type 2). All cases were reviewed independently by two neuropathologists, and the diagnosis was confirmed according to the international consensus classification (Blumcke et al., 2013). As control tissue, we used a specimen from an autopsy of an individual without any neurological disease (#3, Table S1; death by myocardial infarction). The autopsy was performed within 10 to 24 h of death obtaining tissue with preserved immunoreactivity (Roseti et al., 2013). All tissue was snap-frozen over liquid nitrogen and stored at -80 °C until use. Frozen tissue was shipped by courier to University of Rome. Tissue was obtained and used in accordance with the Declaration of Helsinki and the UMC Research Code provided by the Medical Ethics Committee. The Ethics Committee of the University of Rome “Sapienza” and Neuromed I.R.C.C.S approved the technical procedures. Informed consent was obtained from all individuals involved in this study.

**Table S1. Clinical characteristics and neurophysiological findings of MTLE patients**

| P# | Age (yrs)/sex | Epilepsy onset (yrs) | Surgical zone | Seizure type | no. seizures/month | Pathology | Medications | Prognosis after surgery  (follow-up) |
| --- | --- | --- | --- | --- | --- | --- | --- | --- |
| #1 | 36/F | 6 | R-T | FIAS | 10 | HS | CBZ | Seizure free  (five years) |
| #2 | 27/M | 10 | R-T | FIAS | 12 | HS | CBZ, LCS | Seizure free  (six years) |
| #3 | 31/M  control | -- | -- | -- | -- | -- | -- | myocardial infarction |
| #4 | 56/F* | 12 | R-T | FIAS | 2 | HS | CNP | Seizure free  (five years) |
| #5 | 51/M* | 16 | R-T | FIAS | 2 | HS | CBZ, PHB, VGB | Rare disabling seizures (six years) |
| #6 | 29/M* | 4 | L-T | FIAS/GS | 32 | HS | LMT, TPM | Seizure free  (four years) |
| #7 | 48/M* | 30 | L-T | FAS | 32 | HS | LMT,VPA | Seizure free  (five years) |
| #8 | 39/M* | 20 | L-T | FIAS | 14 | HS | CBZ | Seizure free  (six years) |

P#, patients; T, temporal; HS, hippocampal sclerosis, FAS, focal aware seizure; FIAS, focal impaired awareness seizure; GS, generalized seizures; CBZ, carbamazepine; CNP, clonazepam; LCS, lacosamide; LMT, lamotrigine; PHB, phenobarbital; TPM, topiramate; VGB, vigabatrin; VPA, valproic acid; *, tissues used for patch-clamp recordings.

**Electrophysiology**

***Patch-clamp in human slices***

Immediately after surgery, transversal hippocampal slices (350 μm) were cut in glycerol-based artificial cerebro-spinal fluid (ACSF) with a vibratome (Leica VT 1000S; Leica Microsystems); placed in a slice incubation chamber at room temperature with oxygenated ACSF and transferred to a recording chamber within 1–8 h after slice preparation. ACSF had the following composition (in mM): 125 NaCl, 2.5 KCl, 2 CaCl_2_, 1.25 NaH_2_PO_4_, 1 MgCl_2_, 26 NaHCO_3_, 10 glucose, 0.1 Na-pyruvate (pH 7.35; 5% CO_2_).

Whole-cell patch clamp recordings were performed on cells in stratum pyramidale and in stratum oriens, at 24– 25 °C. Pyramidal cells were distinguished from interneurons by localization and morphological properties. Furthermore, the action potential analysis confirmed cell identification, with significantly different after-hyperpolarization values: -4.1 ± 0.6 mV in pyramidal cells and -12 ± 1 mV in interneurons (n=12 and 8, p<0.001). Action potentials (AP) were recorded from neurons applying depolarizing current steps (100-150 pA, 500 ms) using glass electrodes (3-4 MΩ) filled with (in mM): 140 KCl 10 Hepes, 5 BAPTA, 2 Mg-ATP (pH 7.3, with KOH). Data analyzed with ANOVA. For more details, see Ragozzino et al., 2005.

***Membrane Preparation, Injection Procedure, and voltage-clamp Recordings in Oocytes.***

Membrane preparation and injection was performed as already described (Eusebi et al., 2009).

Briefly, human tissues were homogenized using a Teflon glass homogenizer with 2 ml of glycine buffer of the following composition (in mM): 200 glycine, 150 NaCl, 50 EGTA, 50 EDTA, 300 sucrose; plus 20 μl protease inhibitors (Sigma); pH 9 adjusted with NaOH. The homogenate was centrifuged for 15 min at 9.500 x g. The supernatant was collected and centrifuged for 2 h at 10^5^ x g at 4 °C. The pellet was washed, re-suspended in assay buffer (glycine 5 mM) and used directly, or aliquoted and stored at –80 °C for later use.

The rundown of the GABA-evoked currents was elicited by 6 applications of GABA (500 μM) interspaced by 40 s intervals and was defined as the percent decrease of the sixth GABA current peak amplitude (I_GABA_) after five previous applications. Cannabidivarine (CBDV) or brain derived neurotrophic factor (BDNF) were applied as previously described (Ragozzino et al., 2005; Morano et al., 2016) . GABA-current potentiation by cannabidiol (CBD) was tested in previously validated conditions (Ruffolo et al., 2018) and I_GABA_ expressed as a percentage of the currents evoked before drug application. For comparison, in some experiments, we used one control individual that is described in Table S1. In the text, the number of patients used in each experiment is reported and referred to Table S1, using the symbol # and numbers (n) referring either to oocytes or neuronal cells used in each experiment. Animal protocols were approved by the Italian Ministry of Health (authorization no. 78/2015‐PR). From 12 to 48 h after injection, membrane currents were recorded from voltage-clamped *Xenopus laevis* oocytes using two microelectrodes filled with 3M KCl as previously described (Miledi et al., 2006). The oocytes were placed in a recording chamber (0.1ml volume) and perfused continuously with oocyte Ringer solution (OR in mM: 82.5 NaCl; 2.5 KCl; 2.5 CaCl_2_; 1 MgCl_2_; 5 Hepes, adjusted to pH 7.4 with NaOH) at room temperature (20-22°C). To apply GABA or OR we used a gravity driven multi-valve perfusion system (9-10 ml/min) controlled by computer (Biologique RSC-200; Claix, France) to ensure the exact duration of each application. Using this system, 0.5 to 1s are sufficient to reach the complete replacement of applied solution. GABA current rundown was defined as the percentage decrease of the current peak amplitude after six 10s-applications of GABA at 40s intervals (Eusebi et al., 2009; Roseti et al., 2013). All the salts were purchased by Sigma Aldrich (USA), GABA was purchased by Tocris Bioscience (Bristol, UK) while cannabis derivatives were purchased by THC Pharma (Frankfurt, Germany). BDNF (Sigma) was dissolved in H_2_O, stored as frozen stock solutions (50 μg/ml) until use. CBD and CBDV were dissolved in ethanol and then diluted to the desired concentration in OR. The final dilution of ethanol was always lower than 1:5000.

**Table S2. Electrophysiological parameters in patch-clamped human neurons**

|  | MTLE pyr | Na_V_1.1 pyr | MTLE int | Na_V_1.1 int |
| --- | --- | --- | --- | --- |
| resting membrane potential (mV) | -68 ±1 | -68 ± 2 | -74 ± 2 | -63 ± 8 |
| AP half width (ms) | 2.3±0.4 | 2.2±0.4 | 1.6±0.2 | 1.9±0.3 |
| afterhyperpolarization (mV) | -4.1 ± 0.6 | -4.0 ± 1 | -11 ± 2 | -13 ± 2 |

Data represent means ± SEM; AP, action potential; pyr, pyramidal cells; int, interneurons.

**Table S3. Effects of pharmacological agents on mNa_V_1.1 and MTLE patients**

| **patients** | **I_GABA_ rundown (%)** | | | | **I_GABA_ increase (%)** | | |
| --- | --- | --- | --- | --- | --- | --- | --- |
|  | control | +CBDV 50 nM | *n* | +BDNF 0.5μg/ml | *n* | +CBD 5 μM | *n* |
| **mNav 1.1** | 43.2+3.1 | 71.0+5.5 p<0.01 | 19 | 72.5+3.2 p<0.01 | 8 | +29.8+4.1 p<0.01 | 13 |
| **MTLE** | 49.0+9.1 | 70.1+5.7 p<0.01 | 33 | 74.7+4.5 p<0.01 | 9 | +27.5+8.8 p<0.01 | 22 |

Data represent means ± SEM; *n*, number of cells; CBDV, cannabidivarine; CBD, cannabidiol.

GABA concentration was 500 μM in rundown experiments, and 50 μM in CBD experiments.

**
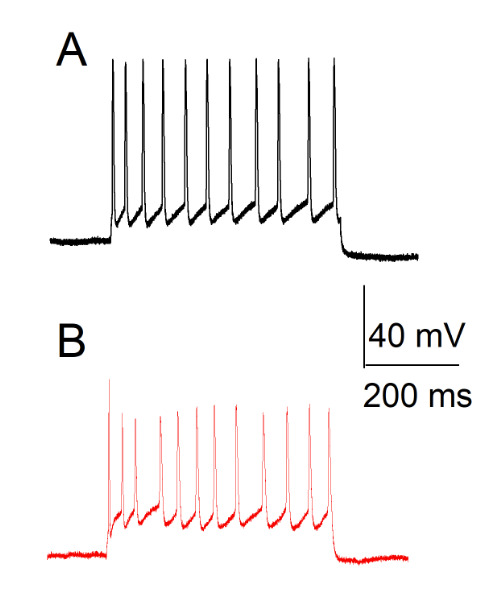
**

**Figure S1. *Firing of hippocampal interneurons***

**A**, typical trace showing action potential (AP) properties in a current-clamped MTLE hippocampal interneuron. Current intensity, 100 pA. **B**, typical trace showing AP properties in a current-clamped mNa_V_1.1 hippocampal interneuron.

**Statistics**

Before data analysis, normal distribution was assessed with Shapiro-Wilk test. According to the result parametric (Student's t-test; ANOVA and post hoc Holm–Sidak test), or non-parametric (Wilcoxon signed rank test, Mann-Whitney rank sum test) tests have been used. The statistical analysis of the data was performed with Sigmaplot 12 software, and differences between two data sets were considered signiﬁcant when p < 0.05.

**Supplementary References**

1. Blumcke I, Cross JH, and Spreafico R. The international consensus classification for hippocampal sclerosis: an important step towards accurate prognosis. Lancet Neurol 2013; 12, 844–846.
2. Roseti C, Fucile S, Lauro C, et al. Fractalkine/CX3CL1 modulates GABAA currents in human temporal lobe epilepsy. Epilepsia 2013; *54*, 1834–1844.
3. Miledi R, Palma E, and Eusebi F. Microtransplantation of neurotransmitter receptors from cells to Xenopus oocyte membranes: new procedure for ion channel studies. Methods Mol. Biol 2006; 322, 347–355.
4. Eusebi F, Palma E, Amici M, et al. Microtransplantation of ligand-gated receptor-channels from fresh or frozen nervous tissue into Xenopus oocytes: a potent tool for expanding functional information. Prog. Neurobiol. 2009; 88, 32–40.
5. Ragozzino D, Palma E, Di Angelantonio S, et al. Rundown of GABA type A receptors is a dysfunction associated with human drug-resistant mesial temporal lobe epilepsy. Proc Natl Acad Sci U S A. 2005;102, 15219–15223.
6. Eusebi F, Palma E, Amici M, et al. Microtransplantation of ligand-gated receptor-channels from fresh or frozen nervous tissue into Xenopus oocytes: a potent tool for expanding functional information. Prog. Neurobiol. 2009; 88, 32–40.
7. Morano A, Cifelli P, Nencini P, et al. Cannabis in epilepsy: From clinical practice to basic research focusing on the possible role of cannabidivarin. Epilepsia Open 2016; 1, 145–151.
8. Ruffolo G, Cifelli P, Roseti C, et al. A novel GABAergic dysfunction in human Dravet syndrome. Epilepsia 2018; 59, 2106–2117.
